# Visualizing a Cold Stress-Specific Pulse Wave in Traditional Pulse Diagnosis (‘Tight Pulse’) Correlated with Vascular Changes in the Radial Artery Induced by a Cold Pressor Trial

**DOI:** 10.3390/s24072086

**Published:** 2024-03-25

**Authors:** Jichung Song, Jae Young Choi, Byung-Wook Lee, Dongmyung Eom, Chang-Hyun Song

**Affiliations:** 1 Department of Medical History, College of Korean Medicine, Daegu Haany University, Gyeongsan 38610, Republic of Korea; prunedias@gmail.com; 2 Department of Urology, College of Medicine, Yeungnam University, Daegu 42415, Republic of Korea; urocjy@ynu.ac.kr; 3 Department of Medical Classics & History, College of Korean Medicine, Dongguk University, Gyeongju 38066, Republic of Korea; omis@dongguk.ac.kr; 4 Department of Medical Classics, College of Korean Medicine, Wonkwang University, Iksan 54538, Republic of Korea; haksan@wku.ac.kr; 5 Department of Anatomy and Histology, College of Korean Medicine, Daegu Haany University, Gyeongsan 38610, Republic of Korea

**Keywords:** cold stress, ice cube test, pulse diagnosis, tight pulse, TCM, TEAM, ultrasonography

## Abstract

Radial pulse diagnosis is the most common method to examine the human health state in Traditional East Asian Medicine (TEAM). A cold stress-related suboptimal health state (subhealth) is often undetectable during routine medical examinations, however, it can be detected through the palpation of specific pulse waves, particularly a ‘tight pulse’, in TEAM. Therefore, this study examined a correlation between ‘tight pulse’ and vascular changes in the radial artery (RA) induced by a cold pressor trial (CPT). Twenty healthy subjects underwent sequentially control trial and CPT with room-temperature and ice-cold water, respectively, on the right forearm. The radial pulse and vascular changes were then examined on the left arm. The radial pulse scores for frequencies of ‘tight pulse’ with strong arterial tension increased after the CPT compared with the control trial. The pulse scores were reversely correlated with the RA thickness and volumes in ultrasonography, but not with changes in the systolic/diastolic blood pressure. The RA thickness-based vascular surface and three-dimensional images visualized a ‘tight pulse’ showing the vasoconstriction and bumpy-/rope-shaped vascular changes in the radial pulse diagnostic region after the CPT. These findings provide valuable insights into the potential integration of clinical radial pulse diagnosis with ultrasonography for cold-related subhealth.

## 1. Introduction

Radial pulse palpation has been most commonly used to diagnose the human health state in Traditional East Asian Medicine (TEAM) based on Traditional Chinese Medicine (TCM) [1,2,3]. Radial pulse diagnosis is performed by palpating above the wrist joint using fingertip sensing to examine the pulse size, rate, and rhythm on the radial artery (RA), approximately 3–5 cm in length [1,4]. Normal pulse waves are classically described as ‘spirited’ and ‘neither hard and unyielding nor flaccid and indistinct’, with an average speed of 70–75 beats per minute, while the abnormal changes can be expressed as 28 pulse conditions, such as heat-induced ‘rapid pulse’, fatigue-induced ‘string pulse’, and Qi stagnation-induced ‘rough pulse’ [5]. However, the traditional pulse theory is ambiguous in its description, and it requires scientific reevaluations for the optimal diagnosis in quite different modern lifestyles and health statuses from those of previous eras.

Suboptimal health status (subhealth) is caused by some disturbances in psychological behaviors or physical function and adaptation capacity; however, it often remains undetectable through hospital examinations due to the absence of typical pathological characteristics [6]. The symptoms include fatigue, headaches, indigestion, nausea, insomnia, irritability, forgetfulness, and lack of concentration [7]. Exposure of the human body to external cold stress augments peripheral vasoconstriction and blood pressure via a sympathoexcitatory pathway to maintain thermal homeostasis [8]. Cold stress often induces subhealth by disrupting the balance of endogenous and cardiovascular systems, potentially leading to various cardiovascular diseases (e.g., stroke and coronary vascular disease) [9,10,11]. The cardiovascular and catecholamine responses indeed persist long even after the cold stress has ceased [12]. In TEAM, individuals exposed to a cold environment are diagnosed by radial pulse palpation with a ‘tight pulse’, ‘short pulse’, and ‘fine or small pulse’ [13,14,15]. Among them, ‘tight pulse’ may serve as a specific diagnostic indicator for cold-related subhealth, because ‘short pulse’ and ‘fine or small pulse’ can also present in other health conditions with deficiencies in nutrients or Qi circulation and fragility or lethargy, respectively [16]. ‘Tight pulse’ is described as ‘string’, ‘rope’, or ‘cord’ in the WHO International Standard Terminologies on Traditional Medicine and TEAM [14]. Although the radial pulse diagnosis can provide earlier warnings of physiological processes in the subhealth, it is difficult to understand ‘tight pulse’ in modern medical terms. In addition, the subjective pulse diagnosis can vary among TEAM practitioners and within the same practitioner as inter- and intra-practitioner reliability, respectively. This underscores the urgent need for objective quantification and visualization of the specific pulse wave patterns [17]. 

Some pulse sensor devices have been developed for objective radial pulse diagnosis [1,18,19,20]. In addition, there have been various attempts aimed at achieving the following objectives: identifying the optimal diagnostic region for radial pulse palpation using ultrasonographic imaging [21]; developing an optimal method using spatial-carrier digital speckle pattern interferometry [22]; assessing the associations between pulse waves and hemodynamics in hypertensive patients [23]. Furthermore, recent studies have interpreted radial pulse diagnosis by measuring the intima and media thickness of arteries using power spectral analysis in pregnant subjects [24] and patients with dyspepsia, rhinitis [25], atopic dermatitis [26], and hypertension [27]. However, disease-specific pulse conditions and the actual pulse shapes remain unclear. Therefore, we examined correlations between cold stress-specific ‘tight pulse’ and vascular changes in the RA induced by a cold pressor test (CPT) and visualized the pulse wave patterns.

## 2. Materials and Methods

### 2.1. Subjects

This study protocol was approved by the Institutional Review Board of Daegu Haany University (Gyeongsan, Republic of Korea, No. DHUMC-D-20012-PRO-02) and registered from Korea Disease Control and Prevention Agency (No. PRE20220629-002). Twenty-five subjects were recruited through posters at Daegu Haany University. After obtaining written informed consent, the subjects received physical examinations, including body weight, height, body mass index (BMI), body surface area (BSA), and systolic/diastolic blood pressure (SBP/DBP). BMI and BSA were calculated using the following Formulas (1) and (2) [28]:

BMI (kg/m^2^) = (body weight, kg)/(height, m)^2^, (1)

BSA (m^2^) = square root [(weight, kg, × height, cm)/3600]. (2)

Blood pressure was measured using a standard mercury sphygmomanometer in a quiet and temperature-controlled room (22–24 °C). Exclusion criteria included obesity (BMI ≥ 30 kg/m^2^), hypertension (SBP/DBP > 140/90 mmHg) [29], a history of cardiovascular diseases or cold-mediated anaphylaxis, and the use of antihypertensive or analgesic medications within the past 6 weeks. Five subjects were excluded due to obesity (*n* = 2), hypertension (*n* = 1), and records of other treatments (*n* = 2), and a total of 20 healthy normotensive subjects were enrolled for further sessions, scheduled at 2–6 p.m. The subjects were instructed to maintain their regular diets, but abstain from heavy meals, nicotine, caffeine, or alcohol consumption 24 h before the sessions.

### 2.2. Study Design

The subjects underwent sequential trials of control trial and CPT at 1 h-interval between them, as described elsewhere [30,31]. The control trial and CPT were performed by placing a thermocol bag containing 1 L of room-temperature water and cold water mixed with ice cubes, respectively, applied to the right proximal forearm for up to 5 min. The radial pulse score, ultrasonography of RA, and SBP/DBP were examined on the contralateral left arm in a comfortable posture on a fixed splint, prior to the trial as the baseline and each after the sequential trials. The radial pulse changes were scored in the first session, and the ultrasonography of RA and SBP/DBP were assessed in the second session. Subjects had a resting period for a week between the sessions.

### 2.3. Radial Pulse Diagnosis and Ultrasonography

Radial pulse diagnosis was performed by gently pressing the RA against the underlying bone above the wrist joint, near the base of the thumb, using the index, middle, and ring fingers. The changes of pulse wave were scored from 0 to 4 based on the frequencies of ‘tight pulse’ with strong arterial tension by three TEAM practitioners blinded to the study design, as follows: 0, no ‘tight pulse’; 1, faint ‘tight pulse’; 2, some ‘tight pulse’ with weak arterial tension; 3, some ‘tight pulse’ with slightly increased arterial tension; 4, bounding ‘tight pulse’ with strong arterial tension. The vascular changes of RA were examined using medical ultrasonography (LOGIQ^TM^ 5 Basic; GE Healthcare, Wauwatosa, WI, USA). The RA thickness was measured in the pulse diagnostic region or 20 arterial segments at 1 mm intervals from 12 mm proximal to 8 mm distal to the diagnostic region. Half of the thickness values were designated as a radius of the arterial segments, and the connected line was used for creating a three-dimensional image using Adobe Illustrator CS6 software version 16.0. The segmental volume of RA was calculated as the following Formula (3):

Volume = ⅓πh × (R_1_^2^ + R_1_ × R_2_ + R_2_^2^), (3)

(where ‘R_1_’ and ‘R_2_’ represent the radii of both terminal ends in each segment, and ‘h’ is set at 1 mm as the segmental interval). The total RA volume across all segments of 20 mm in length was determined by summing the segmental volumes.

### 2.4. Statistical Analysis

All data are represented as the means ± standard errors of the means (SEMs). The normal distribution of the variables was determined by the Shapiro–Wilk test with 95% confidence. The bivariable correlation was examined to quantify the strength of associations among the parameters by the Pearson and Spearman correlation coefficients for normally and non-normally distributed data, respectively. In the subjects’ baseline characteristics, the correlations were analyzed mainly in the cardiovascular parameters including the body temperature, heart rate, and SBP/DBP. The time- and trial-dependent sequential data were examined by the Paired-sample *t*-tests between two consecutive time-points or among the baseline and sequential trials. Statistical significance was set at *p* < 0.05.

## 3. Results

### 3.1. Subjects’ Baseline Characteristics

A total of 20 subjects (12 males and 8 females) participated in this study, and their baseline characteristics are listed in Table 1. There were significant correlations among the SBP, DBP, and heart rate (*p* < 0.05). The SBP was also correlated with the genders, body weight, BMI, and BSA (*p* < 0.05), indicating that the SBP was higher in the males than females, and proportional to the DBP, heart rate, body weight, BMI, and BSA. In addition, significant correlations were found among the genders, body weight, height, BMI, and BSA, except between height and BMI (*p* < 0.01). The body weight, height, BMI, and BSA were higher in males than females; the body weight was proportional to the height, BMI, and BSA; the BSA was proportional to the height and BMI. However, the subjects’ age and body temperature had no correlations with the others.

### 3.2. Traditional Radial Pulse Scores

**Figure 1:**
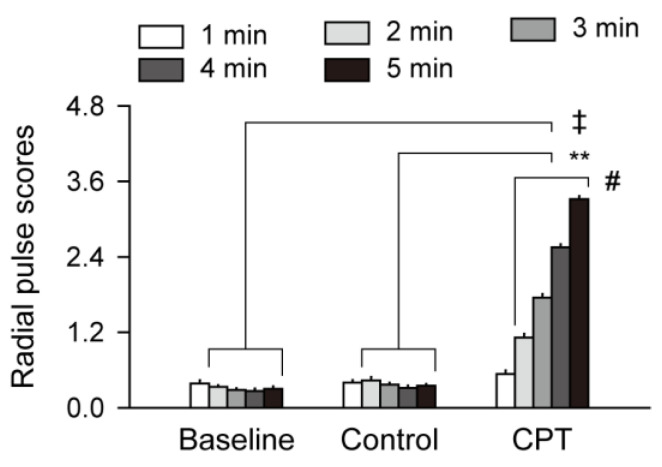
Time-dependent radial pulse scores. The radial pulse changes were scored at 1 min-interval for 5 min, prior to the trial (baseline) and each after the control and cold pressor trial (CPT). Values are represented as the means ± standard errors of the means (SEMs). ‡ and **: *p* < 0.01 vs. the baseline and control trial, respectively, and #: *p* < 0.01 between the consecutive time-points after the CPT, by the Paired-sample *t*-tests.

All subjects underwent sequential trials of the control trial and CPT, on the right forearm for up to 5 min, and the radial pulse changes were scored on the left arm every minute for 5 min, in the baseline and each after the sequential trials (Figure 1). Since the pulse scores were significantly correlated with the trials (*p* < 0.01), but not with the genders and ages, they were analyzed in the combined data from both genders of all ages at each time-point. There were no differences in the pulse scores for 5 min between the baseline and control trials. However, the pulse scores were time-dependently increased at two to five min after the CPT, and they were higher at the same time-points after the CPT than in the baseline and after the control trial (Pared-sample *t*-tests, *p* < 0.01).

### 3.3. Vascular Changes in the Radial Pulse Diagnostic Region

**Figure 2:**
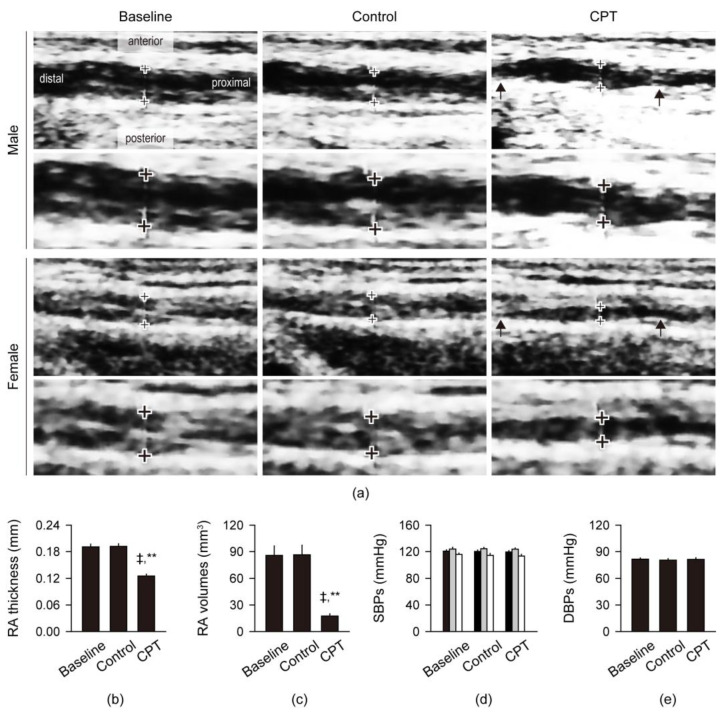
Ultrasonography of the radial artery and blood pressure. (**a**) Representative images of the radial artery (RA). The pulse diagnostic regions (+) were high-magnified in lower in the males and females. Arrows indicate evident vasoconstriction of the RA; (**b**,**c**) RA thickness and volumes in the pulse diagnostic region; (**d**,**e**) Systolic/diastolic blood pressures (SBPs/DBPs). Black, gray, and white bars indicate the values in the total subjects, males and females. Values are represented as the means ± SEMs. ‡ and **: *p* < 0.01 versus the baseline and control trial, respectively, by the Paired-sample *t*-tests.

One week later, the subjects underwent the same sequential trials on the right forearm for 5 min, and the vascular changes were examined by the ultrasonography of RA and SBP/DBP on the left arm (Figure 2). In ultrasonography, the vascular changes of RA were little different between the baseline and control trials (Figure 2a). However, the RA around the pulse diagnostic region exhibited evident vasoconstriction and irregular vascular surfaces that were extremely constricted in the proximal and distal to the diagnostic region. There were significant correlations among the trial-dependent radial pulse scores and RA thickness and volumes in the pulse diagnostic region (*p* < 0.01). Although significant correlations were also found between the genders and SBPs and between the SBPs and DBPs (*p* < 0.05), the trial-dependent radial pulse scores and RA thickness and volumes had no correlations with the genders, ages, and SBPs/DBPs. The RA thickness and volumes in the pulse diagnostic region were not different between the baseline and control trial; however, they were significantly reduced after the CPT compared with the baseline and control trial (Paired-sample *t*-tests, *p* < 0.01, Figure 2b,c). The RA thickness and volumes were 100.5% and 100.8%, respectively, after the control trial compared with the baseline, while they were reduced by 65.4% and 20.4%, respectively, after the CPT compared with the control trial. The SBPs/DBPs were not different among the baseline and sequential trials in both genders and total subjects (Figure 2d,e).

### 3.4. Three-Dimensional Images of the Radial Artery around the Pulse Diagnostic Region

**Figure 3:**
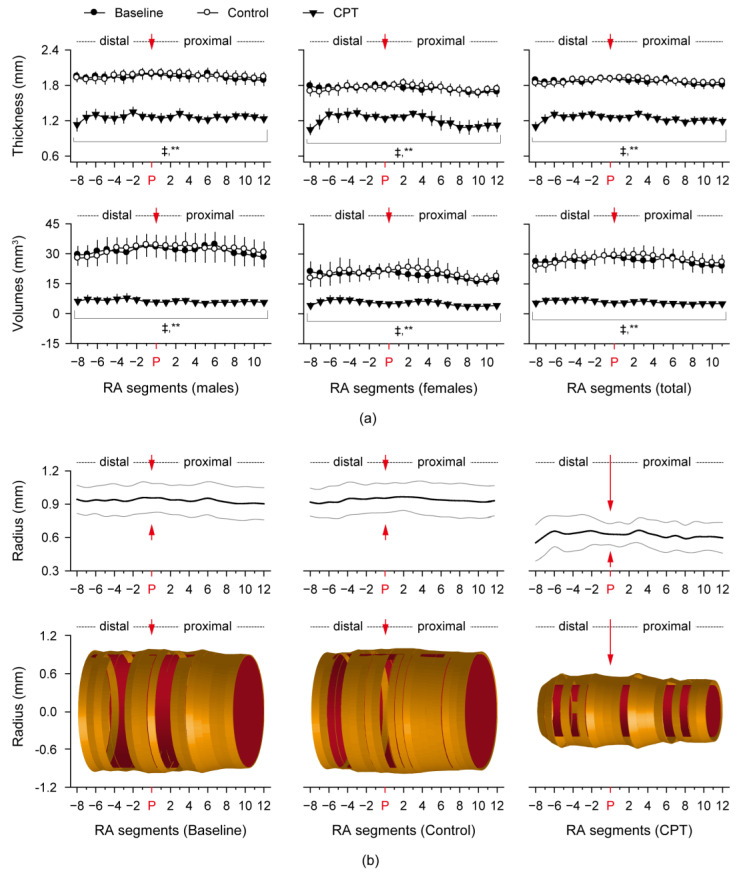
Three-dimensional images of ‘tight pulse’ around the radial pulse diagnostic region. (**a**) RA Thickness (upper) and volumes (lower) in 20 arterial segments at 1 mm-interval from 12 mm proximal to 8 mm distal to the pulse diagnostic region. (**b**) RA thickness-based radius (upper) and the three-dimensional images (lower). Black and gray lines indicate the mean radii and SEMs, respectively. Red ‘P’ and arrow indicate the pulse diagnostic region. Values are represented as the means ± SEMs. ‡ and **: *p* < 0.01 versus the baseline and control trial, respectively, by the Paired-sample *t*-tests.

Ultrasonographic images of the RA were further analyzed in 20 arterial segments divided at 1 mm interval from 12 mm proximal to 8 mm distal to the radial pulse diagnostic region (Figure 3). There were significant correlations among the trial-dependent vascular changes on the RA thickness and volumes (*p* < 0.01). Although the vascular changes were also correlated with the genders in the baseline and after the control trial (*p* < 0.01), the trial-dependent vascular changes had no correlations with the genders and ages. While the RA thickness and volumes at each segment were not different between the baseline and control trial, they were significantly reduced in both genders and total subjects, after the CPT compared with the baseline and control trial (*p* < 0.01, Figure 3a). The volumes at all RA segments of 20 mm in length were 103.0% in the subjects (102.1% in the males and 105.2% in the females), after the control trial compared to the baseline. Notably, the volumes were significantly reduced by 21.5% in the subjects (19.3% in the males and 26.5% in the females), after the CPT compared to the control trial (*p* < 0.01). The RA thickness-based vascular surfaces and three-dimensional images visualized a ‘tight pulse’ showing the vasoconstriction and bumpy-/rope-shaped vascular changes, in the radial pulse diagnostic region after the CPT compared with the baseline and control trial (Figure 3b).

## 4. Discussion

This study is the first to demonstrate significant correlations between ‘tight pulse’ and vascular changes in the RA induced by CPT and visualize the specific pulse wave pattern. The subjects’ baseline characteristics were consistent with those reported in other studies [32]. For example, the SBPs and pulse waves were higher and more forceful in the males than in females [33]; however, the radial pulse scores and vascular changes showed similar trends in both genders after the CPT compared with the baseline and control trials. The generation of a ‘tight pulse’ was reversely correlated with the RA thickness and volumes in the pulse diagnostic region, but not with changes of the SBP/DBP. This suggests that the ‘tight pulse’ may result from the localized fine vasoconstriction through signal transduction in the RA, rather than systemic vascular responses. Radial pulse pressure is derived from the heart’s pumping action and transmitted to the end of RA as a muscular artery [15]. In response to stress conditions, sympathetic nervous activity promptly reacts to increased vessel impedance by releasing neurotransmitters including acetylcholine, which directly induces vasoconstriction in peripheral arteries [34]. Additionally, if catecholamines sustain the long-term stability of cardiovascular responses to cold stress, acetylcholine can be released due to parasympathetic stimulation, leading to acetylcholine-dependent muscle contraction [8,12]. Previous studies have reported that CPT induces long-term stability in cardiovascular and catecholamine responses [8,35]. The pulse waves serve as a driving force for the pulses and vasomotion while simultaneously providing information about the velocity and shape of blood flow [15]. Here, the CPT-induced vasoconstriction produced a bumpy- or rope-shaped ‘tight pulse’ with strong arterial tension, which may be involved in irregular blood flow velocity caused by the sustained vascular constriction in the RA segments.

Radial pulse diagnosis is the most common diagnostic method in TEAM because of its convenience, cost-effectiveness, and non-invasiveness. The pulse diagnosis can be also assessed at the carotid, temporal artery, dorsalis pedis, and inguinal arteries [15]. However, the RA serves as the main region to diagnose the balance of Qi and blood and assess the homeostasis of the human body and organ systems [1,2,3]. The reason is that the radial pulse is minimally affected by aging, arterial pressure, or various motions, making it more reliable compared to the pulse waves of other arterial vessels. In addition, pulse sensing is relatively superficial at the RA supported by the radial styloid process and tendons, in contrast to other arteries surrounded by soft tissues [36]. The cold stress-induced pulse wave signals are known to produce ‘tight-, short-, and fine or small-pulses’. The ‘tight pulse’ can be a primary indicator of subhealth related to the cold and pain, while ‘short- and fine or small-pulses’ can also present in cases of deteriorated health conditions [13,14,15]. Indeed, the current CPT induced mainly a ‘tight pulse’ probably due to responses to cold stress in healthy subjects. However, a ‘tight pulse’ may also present in internal obstructive disorders related to poor digestion, or gradually change to a ‘firm pulse’ followed by a ‘hidden pulse’ according to the chronic progression of cold from the external to interior body regions [14,15]. In this context, differential diagnoses are necessary to discern the causes of cold-induced pulse changes by assessing other signs and symptoms in gastroscopy and ultrasonography, or routine medical examinations including temperature, heart rate, and blood pressure.

Cold stress-related subhealth often goes undetected during routine hospital examinations, despite its potential link to the elevation of risk factors for various cardiovascular diseases. Previously, hypertension has been reported to have significant correlations with the maximum amplitudes of string-like ‘tight pulse’ [37]. Although some pulse sensor devices have been developed to detect floating/sunken and vacuous/replete pulses on the wrist’s RA to visualize the pulse waves, linking intuitive pulse sensing to objective assessment under certain subhealth has proven challenging [38]. Indeed, most TEAM practitioners still tend to rely on traditional pulse diagnostic methods rather than pulse diagnostic devices. These findings imply that traditional radial pulse diagnosis combined with ultrasonography could offer a promising diagnostic approach for identifying a ‘tight pulse’ involved in cold-related subhealth. However, this study has several limitations as follows: the measurements of radial pulse and vascular changes were taken soon after the CPT, and the trials were performed in small sample sizes of subjects with a limited range of ages. It suggests that the ‘tight pulse’ may serve as an early stage diagnostic signal for cold-induced subhealth at least. Given the frequent occurrence of extreme variations in pulse wave patterns in clinical patients with imbalanced *Qi* or blood flow [39], future clinical studies with large sample sizes are required to explore the disease-specific ‘tight pulse’.

## 5. Conclusions

The CPT-induced radial pulse changes were correlated with vasoconstriction in the local diagnostic region of the radial artery, rather than the systemic vascular changes of SBP/DBP. The vascular surface in the diagnostic region exhibited specific ‘tight pulse’-like changes in ultrasonography after the CPT. It suggests that radial pulse palpation combined with ultrasonography may provide the earliest warnings of physiological processes in the subhealth that may develop into the subsequent disease. This approach is expected to assist in guiding clinical diagnosis for a cold-related subhealth and understanding ‘tight pulse’ for educational purpose in TEAM.

## Figures and Tables

**Table 1:** Subjects’ baseline characteristics.

No.	Gender	Age	BW (kg)	Height (cm)	BMI (kg/m^2^)	BSA (m^2^)	Temp (°C)	HR (bpm)	BP (mmHg)	
									SBP	DBP
1	Male	21	63.5	176.8	20.3	1.8	36.5	69	105	72
2	Male	21	70.0	178.0	22.1	1.9	36.4	94	124	85
3	Male	23	72.0	174.3	23.7	1.9	36.4	78	127	88
4	Male	24	80.8	186.6	23.2	2.1	36.6	84	114	82
5	Male	26	82.0	178.0	25.9	2.0	36.2	96	130	78
6	Male	27	86.5	172.1	29.2	2.0	36.6	89	113	78
7	Male	31	62.0	165.0	22.8	1.7	36.5	63	120	75
8	Male	31	95.0	179.8	29.4	2.2	36.7	87	121	78
9	Male	32	79.0	174.0	26.1	2.0	36.8	92	125	82
10	Male	33	80.0	176.0	25.8	2.0	36.2	80	123	80
11	Male	39	73.0	174.5	24.0	1.9	36.2	82	126	82
12	Male	49	82.0	175.0	26.8	2.0	36.6	88	128	84
13	Female	23	47.2	167.3	16.9	1.5	36.6	76	108	76
14	Female	23	46.0	158.9	18.2	1.4	36.9	87	118	86
15	Female	23	65.8	177.3	20.9	1.8	36.8	82	116	85
16	Female	23	55.0	157.5	22.2	1.6	36.2	85	111	74
17	Female	25	50.0	170.0	17.3	1.5	36.9	81	122	80
18	Female	26	50.0	167.0	17.9	1.5	36.4	86	112	80
19	Female	34	55.0	169.0	19.3	1.6	36.6	62	106	65
20	Female	43	62.0	163.0	23.3	1.7	36.6	73	115	73
Males (*n* = 12)		30 ± 2	77.2 ± 2.7	175.8 ± 1.5	24.9 ± 0.8	1.9 ± 0.0	36.5 ± 0.1	84 ± 3	121 ± 2	80 ± 1
Females (*n* = 8)		28 ± 3	53.9 ± 2.5	166.3 ± 2.3	19.5 ± 0.8	1.6 ± 0.0	36.6 ± 0.1	79 ± 3	114 ± 2	77 ± 2
Total (*n* = 20)		29 ± 2	67.8 ± 3.2	172 ± 1.6	22.8 ± 0.8	1.8 ± 0.0	36.5 ± 0.1	82 ± 2	118 ± 2	79 ± 1

Values are represented as the means ± SEMs in both genders and total subjects. BMI: body mass index; bpm: beats per minute; BSA: body surface area; BP: blood pressure; BW: body weight; HR: heart rate; SBP/DBP: systolic/diastolic BP; Temp: temperature.

## Data Availability

Data are contained within the article.

## References

[1] Wang H.-Y., Zhang P.-Y. (2008). A model for automatic identification of human pulse signals. J. Zhejiang Univ.-Sci. A.

[2] Walsh S., King E. (2008). Pulse Diagnosis-A Clinical Guide.

[3] Hammer L.I. (2005). Chinese Pulse Diagnosis: A Contemporary Approach.

[4] Birch S. (1992). Naming the unnameable: A historical study of radial pulse six position diagnosis. Trad. Acupunct. Soc. J..

[5] Kaptchuk T.J. (2000). The Web That Has No Weaver: Understanding Chinese Medicine.

[6] Kung Y.Y., Kuo T.B.J., Lai C.T., Shen Y.C., Su Y.C., Yang C.C.H. (2021). Disclosure of suboptimal health status through traditional Chinese medicine-based body constitution and pulse patterns. Complement. Ther. Med..

[7] Li G., Xie F., Yan S., Hu X., Jin B., Wang J., Wu J., Yin D., Xie Q. (2013). Subhealth: Definition, criteria for diagnosis and potential prevalence in the central region of China. BMC Public Health.

[8] Zhang M., Zhao Q., Mills K.T., Chen J., Li J., Cao J., Gu D., He J. (2013). Factors associated with blood pressure response to the cold pressor test: The GenSalt Study. Am. J. Hypertens..

[9] Polcaro-Pichet S., Kosatsky T., Potter B.J., Bilodeau-Bertrand M., Auger N. (2019). Effects of cold temperature and snowfall on stroke mortality: A case-crossover analysis. Environ. Int..

[10] Luo B., Zhang S., Ma S., Zhou J., Wang B. (2012). Effects of cold air on cardiovascular disease risk factors in rat. Int. J. Environ. Res. Public Health.

[11] Gao Z., Wilson T.E., Drew R.C., Ettinger J., Monahan K.D. (2012). Altered coronary vascular control during cold stress in healthy older adults. Am. J. Physiol. Heart Circ. Physiol..

[12] Pettit S.E., Marchand I., Graham T. (1999). Gender differences in cardiovascular and catecholamine responses to cold-air exposure at rest. Can. J. Appl. Physiol..

[13] Wang Y.-Y.L., Wang S.-H., Jan M.-Y., Wang W.-K. (2012). Past, Present, and Future of the Pulse Examination (脈診 mài zhěn). J. Tradit. Complement. Med..

[14] Hsiu H., Huang S.-M., Hsu C.-L., Hu S.-F., Lin H.-W. (2012). Effects of cold stimulation on the harmonic structure of the blood pressure and photoplethysmography waveforms. Photomed Laser Surg..

[15] Walsh S., King E. (2007). Pulse Diagnosis E-Book: A Clinical Guide.

[16] Tang A.C.Y. (2012). Review of traditional Chinese medicine pulse diagnosis quantification. Complement. Ther. Contemp. Healthc..

[17] Craddock D. (1997). Is Traditional Chinese Medical Pulse Reading a Consistent Practice? A Comparative Pilot Study of Four Practitioners. Bachelor’s Thesis.

[18] Kim H., Kim J.Y., Park Y.J., Park Y.B. (2013). Development of pulse diagnostic devices in Korea. Integr. Med. Res.

[19] Schmittgen T.D., Livak K.J. (2008). Analyzing real-time PCR data by the comparative CT method. Nat. Protoc..

[20] Hu C.S., Chung Y.F., Yeh C.C., Luo C.H. (2012). Temporal and spatial properties of arterial pulsation measurement using pressure sensor array. Evid. Based Complement. Altern. Med..

[21] Kim J.U., Lee Y.J., Lee J., Kim J.Y. (2015). Differences in the Properties of the Radial Artery between Cun, Guan, Chi, and Nearby Segments Using Ultrasonographic Imaging: A Pilot Study on Arterial Depth, Diameter, and Blood Flow. Evid. Based Complement. Altern. Med..

[22] Zhang H., Wu S., Li W., Wang Y., Dong M., Yang L. (2018). Precise Detection of Wrist Pulse Using Digital Speckle Pattern Interferometry. Evid. Based Complement Altern. Med..

[23] Ribeiro de Moura N.G., Cordovil I., de Sá Ferreira A. (2016). Traditional Chinese medicine wrist pulse-taking is associated with pulse waveform analysis and hemodynamics in hypertension. J. Integr. Med..

[24] Liao Y.-T., Chen H.-Y., Huang C.-M., Ho M., Lin J.-G., Chiu C.-C., Wang H.-S., Chen F.-j. (2012). The pulse spectrum analysis at three stages of pregnancy. J. Altern. Complement. Med..

[25] Huang C.M., Chang H.C., Kao S.T., Li T.C., Liao Y.T., Wei C.C., Chen C. (2011). Application of sphygmography to detection of dyspepsia and the rhinitis. Am. J. Chin. Med..

[26] Liou J.-m., Huang C.-M., Chiu C.-C., Wang H.-S., Liao Y.T., Peng Y.-C., Cheng Y.-C., Liang S.-J., Lin J.-G., Chen F.-j. (2011). Differences in pulse spectrum analysis between atopic dermatitis and nonatopic healthy children. J. Altern. Complement. Med..

[27] Huang C.-M., Chang H.-C., Kao S.-T., Li T.-C., Wei C.-C., Chen C.C., Chen F.-j., Tsou S.-S. (2011). Radial pressure pulse and heart rate variability in normotensive and hypertensive subjects. J. Altern. Complement. Med..

[28] Mosteller R.D. (1987). Simplified calculation of body-surface area. N. Engl. J. Med..

[29] Mancia G., Fagard R., Narkiewicz K., Redon J., Zanchetti A., Böhm M., Christiaens T., Cifkova R., De Backer G., Dominiczak A. (2014). 2013 ESH/ESC practice guidelines for the management of arterial hypertension: ESH-ESC the task force for the management of arterial hypertension of the European Society of Hypertension (ESH) and of the European Society of Cardiology (ESC). Blood Press..

[30] Tanaka M., Nakagawa Y., Hayashi M., Kotobuki Y., Katayama I., Fujimoto M. (2020). A case of cold-induced cholinergic urticaria accompanied by cholinergic urticaria showing a positive ice cube test. Allergol. Int..

[31] Visitsuntorn N., Tuchinda M., Arunyanark N., Kerdsomnuk S. (1992). Ice cube test in children with cold urticaria. Asian Pac. J. Allergy Immunol..

[32] Evans J.M., Wang S., Greb C., Kostas V., Knapp C.F., Zhang Q., Roemmele E.S., Stenger M.B., Randall D.C. (2017). Body Size Predicts Cardiac and Vascular Resistance Effects on Men’s and Women’s Blood Pressure. Front. Physiol..

[33] King E., Cobbin D., Ryan D. (2002). The reliable measurement of radial pulse: Gender differences in pulse profiles. Acupunct. Med..

[34] Kinefuchi Y., Fukuyama H., Suzuki T., Kanazawa M., Takiguchi M. (1999). Development of a new catheter-tip pressure transducer. Tokai J. Exp. Clin. Med..

[35] Hassellund S.S., Flaa A., Sandvik L., Kjeldsen S.E., Rostrup M. (2010). Long-term stability of cardiovascular and catecholamine responses to stress tests: An 18-year follow-up study. Hypertension.

[36] Chen C.H., Nevo E., Fetics B., Pak P.H., Yin F.C., Maughan W.L., Kass D.A. (1997). Estimation of central aortic pressure waveform by mathematical transformation of radial tonometry pressure. Validation of generalized transfer function. Circulation.

[37] Lee B.J., Jeon Y.J., Ku B., Kim J.U., Bae J.H., Kim J.Y. (2015). Association of hypertension with physical factors of wrist pulse waves using a computational approach: A pilot study. BMC Complement. Altern. Med..

[38] Kim J.-U., Kim S.-H., Jeon Y.-J., Ryu H.-H., Lee Y.-J., Lee H.-J., Kim J.-Y. (2009). Clinical study of the floating-sinking pulse quantification analysis on ages, left/right, and palpation positions. J. Physiol. Pathol. Korean Med..

[39] Flaws B. (1995). The Secret of Chinese Pulse Diagnosis.

